# Overview of Epitalon—Highly Bioactive Pineal Tetrapeptide with Promising Properties

**DOI:** 10.3390/ijms26062691

**Published:** 2025-03-17

**Authors:** Szymon Kamil Araj, Jakub Brzezik, Katarzyna Mądra-Gackowska, Łukasz Szeleszczuk

**Affiliations:** 1 Department of Organic and Physical Chemistry, Faculty of Pharmacy, Medical University of Warsaw, Banacha 1 Str., 02-093 Warsaw, Poland; szymon.araj@gmail.com (S.K.A.); s088074@student.wum.edu.pl (J.B.); 2 Department of Geriatrics, Faculty of Health Sciences, L. Rydygier Collegium Medicum in Bydgoszcz, Nicolaus Copernicus University in Torun, Skłodowskiej Curie 9 Street, 85-094 Bydgoszcz, Poland; katarzyna.madra@cm.umk.pl

**Keywords:** Epitalon, AEDG, pineal gland, peptide

## Abstract

Epitalon, also known as Epithalon or Epithalone, is a tetrapeptide, Ala-Glu-Asp-Gly (AEDG), which was synthesized based on the amino acids composition of Epithalamin, a bovine pineal gland extract, prior to its discovery in pineal gland polypeptide complex solution. During the last 25 years, this compound has been extensively studied using in vitro, in vivo, and in silico methods. The results of these studies indicate significant geroprotective and neuroendocrine effects of Epitalone, resulting from its antioxidant, neuro-protective, and antimutagenic effects, originating from both specific and nonspecific mechanisms. Although it has been demonstrated that Epitalon exerts, among other effects, a direct influence on melatonin synthesis, alters the mRNA levels of interleukin-2, modulates the mitogenic activity of murine thymocytes, and enhances the activity of various enzymes, including AChE, BuChE, and telomerase, it remains uncertain whether these are the sole mechanisms of action of this compound. Moreover, despite the considerable volume of research on the biological and pharmacodynamic characteristics of Epitalon, the quantity of physico-chemical and structural investigations of this peptide remains quite limited. This review aims to conclude the most important findings from such studies, thus presenting the current state of knowledge on Epitalon.

## 1. Introduction and Basic Information on Epitalon

Over 50 years ago, in 1973, the term Epithalamin was used for the first time in widely accessible scientific research. It describes a pineal polypeptide extract from cattle, which has been found to have unique properties in in vitro and in vivo experiments [1]. The discovery of the bioactivity of this extract and its first research were mainly led by V.K Khavinson and V.N Anisimov [2]. Since then, Epithalamin has been widely studied, including in clinical trials, showing a normalizing effect on the basic functions of the human organism and, as a result, establishing it as a geroprotective agent [3]. Despite the publication of several reviews of Epithalamin [4,5,6,7], to the best of our knowledge, none specifically concentrate on Epitalon.

**Figure 1:**
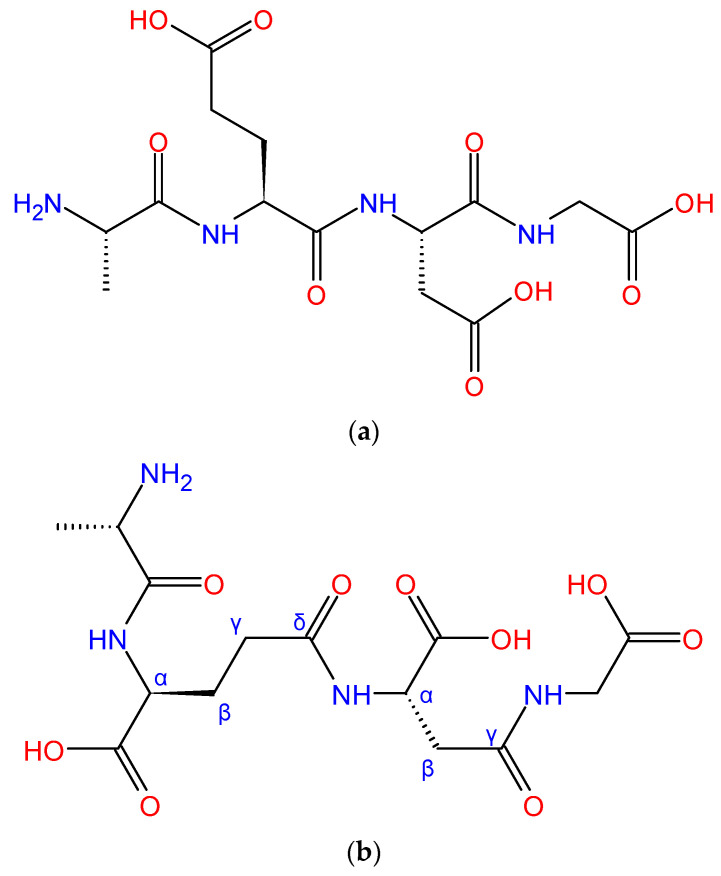
Chemical formula of Epitalon proposed by most authors (**a**) and rarely mentioned “double-gamma bonded” structure (**b**).

Epitalon, also known as Epithalon or Epithalone, is a tetrapeptide that was developed on the basis of the amino acids composition of Epithalamin [8]. For many years, the presence of Epitalon in the human body remained unconfirmed, until 2017, when it was detected for the first time in physiological pineal gland extract [9]. This explains why Epitalon has similar properties to Epithalamin, but differs in the intensity of some actions [10,11]. It should be noted that some authors do not use this common name of Epitalon and refer only to its primary structure—AEDG. This name highlights the fact that Epitalon is a tetrapeptide consisting of Ala-Glu-Asp-Gly (AEDG) amino acids bound through α-peptide bonds (Figure 1a). Interestingly, in one article, where the antioxidant properties of Epitalon were investigated, the authors presented a different chemical structure of Epitalon, composed of the same amino acids but bonded in a different way [12]. In this work, the peptide bond between glutamic acid and aspartic acid was through the δ-carboxylic group and the peptide bond between aspartic acid and glycine was made by the γ-carboxylic group (Figure 1b). The authors named this variant of Epitalon Ala—γ Glu—γ Asp—Gly, which indicates a potential naming mistake. However, this structure of “double-gamma bonded” Epitalon is rather an exception, as we have not encountered it in any other of the published works.

Since Epitalon has a natural origin, it is mostly investigated in its free-base form. However, some authors have specified that Epitalon in the form of salt was used in their experiments. Most common salts of Epitalon available commercially are in combination with acetic or trifluoroacetic (TFA) counterions.

Epitalon, owing to its significant bioactivity and peptide composition, has primarily been studied in vivo through subcutaneous injections. However, some sources assert that Epitalon is a low-hydrolysable peptide that can be taken orally, exhibiting unique features [13]. This study intends to perform a critical evaluation of the published data on Epitalon, encompassing all experimental types—in silico, in vitro, and in vivo. The precise mechanism of action of this tetrapeptide remains unverified, prompting a diversification of the research on both the methodologies employed and the criteria examined. Consequently, we deemed it essential to consolidate the acquired knowledge on Epitalon, not merely as a summary, but also to facilitate the planning of future studies on this interesting peptide.

## 2. Medical, Pharmacological, and Biological Research on Epitalon

Epitalon has been extensively studied in medical, pharmacological, and biological contexts. Our understanding of this chemical is derived from studies conducted on several biological models, which have been utilized and developed to accurately epitomize the results of experiments conducted on humans. To increase the clarity of this review, this chapter is divided into sections based on the biological models employed.

### 2.1. Cells

Epitalon is thought to be a tissue-specific peptide affecting subcortical structures [14]. This was partially confirmed in a study by Brodsky et al., where Epitalon showed no impact on the protein synthesis levels in isolated rat hepatocytes [15]. Also, no significant proliferation rate increase after Epitalon administration was observed in an MTT (3-(4,5-dimethylthiazol-2-yl)-2,5-diphenyltetrazolium bromide) assay experiment conducted on human periodontal ligament stem cells [16].

Despite this, Epitalon’s influence on the proliferation of pigmented and retinal epithelial rat cells has been investigated. Epitalon was compared with Retinalamine at the same concentrations and with the same exposition times. Both compounds induced the active proliferation of the cultured cells. Epitalon showed the best effects after 28 days at a concentration of 10 ng/mL, which, surprisingly, was not the highest concentration used [17]. Following intriguing outcomes from in vivo studies on Epitalon treatment for retinitis pigmentosa [18,19,20], the molecular mechanism of Epitalon was further examined. The expression levels of Pax6, Vsx1, Brn3, Prox 1, and TTR markers were quantitatively assessed and determined to be statistically significantly elevated in isolated cultured chicken retinal tissue treated with an Epitalon- H-Lys(H-Glu-OH)-OH mixture [21].

Epitalon showed also an interesting result in pinealocyte cultured cells originating from rats. The cells were treated with Norepinephrine, Epitalon, and Vilon to check their impacts on melatonin levels. This was achieved by measuring the arylalkyl amine-N-acetyltransferase (AANAT) and cyclic AMP-responsive element-binding protein (pCREB) concentrations in medium post-treatment. The results showed statistically important impacts of Vilon and Epitalon on these two parameters, suggesting a direct effect on melatonin synthesis in pineal cells by these two compounds. The findings of immunohistochemical studies indicated a significant effect of Epitalon on the expression of the pCREB transcription factor and AANAT enzyme in pinealocyte culture. Presumably, Epitalon’s effect on AANAT and pCREB underlies the peptide regulation of pineal cell activity. It is important to note that Epitalon showed a more prolonged mechanism of action than Vilon and was significantly more potent after three hours of the experiment [22]. Djeridane et al. also examined the effect of Epitalon on melatonin release in isolated perifused pineal glands from both young and aged rats. Their experiment demonstrated that the specified tetrapeptide does not influence the melatonin secretion function of the pineal gland. No dependence on the dosage or age of the rats was seen. Furthermore, it was demonstrated that Epitalon did not influence pineal melatonin release stimulated by the β-adrenergic agonist, isoproterenol. The authors proposed that Epitalon did not influence melatonin secretion in vitro [23].

Pineal gland cultures derived from rats have been also used to investigate the effect of Epitalon on lymphocyte differentiation. The experiment’s results indicated that Epitalon decreased the quantity of undifferentiated CD5+ cells, increased the expression of the B-cell marker CD20, and did not influence mature CD4+ and CD8+ T cells. This led Linkova et al. to hypothesize that Epitalon may only enhance the development of lymphocyte precursors into B cells [24]. Another study showed that Epitalon has no impact on the mitotic index of lymphocytes [25].

An additional in vitro experiment conducted on splenocytes derived from CBA mice showed a substantial effect of Epitalon on the mRNA levels of interleukin-2 (IL-2). Epitalon was evaluated against Vilon and Cortagen. The time-dependent efficacy of these peptides was examined by assessing their impact after 5 and 20 h. Epitalon elicited elevated IL-2 mRNA levels just after 5 h, distinguishing it from Vilon, which also affected the splenocytes after 20 h [26]. The effect of Epitalon on mRNA expression led to an experiment performed by L.S Kozina et. al. on human neuroblastoma NB7 cells. The impacts of Epitalon and Vilon on neprilysin (endopeptidase 24.11) (NEP) and insulin-degrading enzyme (EC. 3.4.24.56) (IDE) mRNA were investigated. It was shown that, in normoxia, Epitalon increased the concentrations of IDE and NEP by a statistically insignificant (*p* < 0.05) amount of 10–15%. What is more interesting are the results of the same experiment performed in a hypoxia-culturing environment. In hypoxia, NB7 cells expressed only 70% of IDE and NEP on average. Epitalon was confirmed to fully stop this decrease in mRNA synthesis. This facilitated the revelation of Epitalon’s antihypoxic characteristics concerning NEP and IDP mRNA levels [27].

The previously established mitogenic activity of Epitalon on murine thymocytes [28] served as the foundation for a study by E.I. Grigori’ev, which examined the mechanism of ultra-low doses of this tetrapeptide in aqueous solutions. Epitalon showed the highest activity at concentrations of 10^−17^–10^−15^ M, which are so low that the authors suggested a “Distant reception” dose–activity dependence for Epitalon [29]. Murine thymocytes were also used in a study where potential induced thymocyte blast transformation was investigated. Epitalon did not present such effects in the absence of concavalin A and recombinant interleukin-1β (rIL-1β). A comitogenic effect of Epitalon was observed when thymocytes were co-treated with concavalin A or rIL-1β (*p* < 0.05). Additionally, it was proven that Epitalon does not activate neutral sphingomyelinase. At low concentrations, Epitalon did inhibit this enzyme [28]. Another study where isolated thymocytes were investigated showed that Epitalon has different effects in different stress models [30]. The thymocyte blast transformation reaction was enhanced in the following two stress models: rotational (revolving in containers) and combined (cooling and immobilization). However, proliferation was elevated solely under rotational stress and was inhibited in the combined model. Subsequent analysis concluded that Epitalon modulates IL-1β signal transduction through the sphingomyelin route in cerebral cortex neuron membranes, as evidenced by variations in neutral sphingomyelinase activity in stress models [31].

One experiment revealed a distinctive characteristic of Epitalon, wherein pluripotent cells of *Xenopus laevis* were subjected to various doses of Epitalon. Following a 5-day incubation period, all control cultures (lacking Epitalon therapy) exhibited solely atypical epidermal cells. In cultures with access to Epitalon, the cells were differentiated into epidermal and neural tissue. This impact was found solely at concentrations of 10 ng/mL, 50 ng/mL, and 100 ng/mL. This impact was not detected at concentrations of 2 ng/mL, 20 ng/mL, and 200 ng/mL. The authors of this article did not elucidate the possible rationale for these outcomes [32].

Epitalon also has an effect on telomerase activity. This was confirmed by applying the telomere repeat amplification (TRAP) protocol [33]. The cell cultures used in this experiment were telomerase-positive HeLa cultures and human fetal lung fibroblasts 602/17. Telomerase-positive and Epitalon-treated fibroblasts demonstrated high telomere lengths during the G1 phase of the cell cycle. The authors of this experiment stated that this activity of Epitalon can explain most of the geroprotective effects of this compound [33]. This suggested a potential anti-aging effect which could help to overcome the division limit of human cells in in vitro cultures. This was confirmed by V. K. Khavinson et. al. for human fetal fibroblasts in May 2004. Control fibroblast culture lost its ability for mitosis after the 34th passage. Epitalon-treated cells kept dividing even after the 44th passage, which confirmed the thesis [34]. Another study that used human-originating cells was performed on PHA-stimulated and non-PHA-stimulated lymphocyte cultures. Lymphocytes were taken from healthy participants between 75 and 88 years old, and as a control from donors aged between 25 and 40 years. The first experiment in this study showed a few parameters of chromatin after peptide treatment. Firstly, the amount of associating acrocentric chromosomes per cell was twice as high as in the control group. However, this effect was observed for every peptide treatment in this study and was not specific for Epitalon. Secondly, the dechromatization of chromosomes 1 and 9 for Epitalon treatment was observed. Chromosome 16 stayed stable. The dechromatisation of chromosome 9 only occurred for one other peptide used within this study—Livagen. Lastly, the deheterochromatization level of facultative heterochromatin was observed to be lower in the 75–88-year-old control group compared to the 25–40-year-old one, which was expected. It was experimentally proven that the deheterochromatization level of facultative heterochromatin changed significantly to values higher than those of both control groups after peptide treatment. However, Epitalon was not the most potent peptide [35]. These results were proven again in a similar experiment by Lezhava. et al. in 2023 [36]. A second study was conducted to further evaluate Epitalon’s effects on lymphocyte chromatin. The control group consisted of youthful patients, while senior patients ranged from 71 to 86 years of age. Initially, it was demonstrated that Zinc, Cobalt, and Nickel had a mutagenic effect on chromatin. Subsequently, it was noted that Epitalon exhibits antimutagenic characteristics when co-administered with Zinc, Cobalt, and Nickel [37]. Also, the telomere length after Epitalon administration in PHA-stimulated blood human lymphocytes was investigated. It was found that, in some cases, Epitalon can influence the relative telomere length in young and middle-aged individuals. During this study, individuals with no significant changes, an increased telomere length (in one case. *p* < 0.001 in comparison to controls), and a decreased telomere length (in one case, *p* < 0.01 in comparison to controls) were found. The results of this study indicated that, in human somatic cells, Epitalon can induce the expression of telomerase enzyme components, telomerase activity, and telomere elongation (on average by 33.3%) [25].

**Figure 2:**
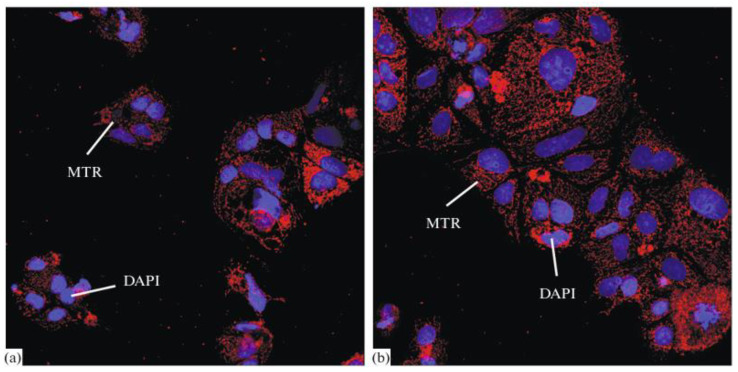
Area of Mito Tracker Red (MTR) staining in old cells of the human pineal gland, immunofluorescence confocal microscopy, magnification of 400×: (**a**) control group and (**b**) AEDG peptide (cell nuclei stained with DAPI-2-(4-Amidinophenyl)-1*H*-indole-6-carboxamidine). Reproduced with permission from Springer Nature [39].

Epitalon was confirmed to have no stimulating effect and more of a suppressing effect on argyrophilic protein expression in thymocytes and thymic epithelial cells from human-aborted embryos cultured simultaneously at all concentrations used within this experiment [38]. Another study conducted in 2020 examined human thymocytes. The impact of Epitalon on the aging of these cells was assessed. The aging process was characterized by a drop in MitoTracker Red mitochondrial labeling, accompanied by a compensatory rise in the synthesis of L7A ribosomal protein. Results were acquired by confocal laser scanning microscopy. Epitalon was again demonstrated to have no impact on the aging processes of thymocytes. However, in this work, human pineal cells were also examined in a similar manner. Epitalon effectively and selectively safeguarded aged human pineal cells from the aging process. The authors concluded that these results validate Epitalon’s tissue-specific activity on the pineal gland, Figure 2 [39].

The expression of other proteins was investigated in vitro. Undifferentiated and neuronic-differentiated human periodontal ligament stem cells were treated with Epitalon. The protein expression of Growth Associated Protein 43 (GAP43) and Nestin was investigated using fluorescent microscopy and Western blotting. Epitalon did increase, but not statistically significantly, the expression rates of these proteins compared to the control group [16]. The next study, where the influence of Epitalon treatment on human gingival mesenchymal stem cells was investigated, was confirmed by RT-PCR, with *p* < 0.01 statistical significancy that Epitalon increases Nestin and GAP43 transcription rates. Additionally, it was found that β-tubulin III and Doublecortin mRNA levels were also significantly influenced by Epitalon administration [40].

The potential ability of Epitalon to traverse the cell membrane was examined. The tetrapeptide was conjugated with fluorescein isothiocyanate, facilitating subsequent analysis. Fodoreyeva et al. conducted several tests with labeled Epitalon on HeLa cell cultures. Initially, the ability of Epitalon to permeate the cell membrane and subsequently enter the nucleus was demonstrated. This suggests that Epitalon’s activity may be associated with cytoplasmic and nuclear components. Further examination concentrated on the interaction of labeled Epitalon, compared to other peptides, with deoxyribo-oligonucleotides tagged with 5,6-carboxyfluorescein. Epitalon was demonstrated to connect with certain DNA sequences, particularly with CAG sequences, which are susceptible to DNA cytosine methylation. This resulted in a hypothesis that Epitalon influences epigenetic cellular activities [41].

Epitalon was observed to enhance the CD4+ population of bone marrow cells, specifically in tissues derived from aged mice [42,43]. An increase in CD8+ cells was observed in the spleen. Elevated levels were seen in the spleen tissue of aged mice. Epitalon also enhances the amounts of thymic serum factor (TSF) in the supernatants of thymic tissue. However, it was shown that other factors, such as melatonin or Epithalamin (non-synthetic Epitalon), had a greater influence on this parameter [44].

Additional features of Epitalon were identified in a study involving its application to SH-SY5Y neuroblastoma cells. The following three criteria were examined: the quantity of amyloid precursor protein and the activity levels of acetyl- and butyrylcholinesterase (AChE and BuChE). Nalieva et al. confirmed an average increase of 10–25% in the activity of AChE and BuChE in the presence of Epitalon in the medium. The authors suggested this phenomenon may have resulted from an elevated secretion of these proteins. This is associated with a reduced activity of AChE and BuChE in the soluble protein fraction treated with Epitalon. An identical effect was noted in an experiment examining membrane-bound variants of AChE and BuChE. Epitalon once more demonstrated a diminishing influence on the activity of these enzymes. The subsequent experiment demonstrated that Epitalon also affected the secretion levels of the soluble form of amyloid precursor protein (APP). The secretion level of this protein increased by 20% following incubation with Epitalon. The authors drew conclusions indicating that Epitalon influences AChE, BuChE, and APP metabolism, positioning it as a possible candidate for the treatment of cholinergic deficiency diseases and cognitive disorders associated with amyloid metabolism [45].

In 2022, a particularly instructive work investigating the influence of Epitalon on human cells was published. This study utilized cell cultures of THP-1 monocytic human leukemia. It was established that Epitalon promotes signal transducer and activator of transcription 1 (STAT1) phosphorylation, potentially through a receptor-independent mechanism that does not include interactions between IFN-α and IFN-R, as seen by the lack of effect on IFN-α production. Furthermore, THP-1 cells exposed with bacterial-derived lipopolysaccharide (LPS) exhibited an additive effect on the tyrosine phosphorylation of extracellular signal-regulated kinases 1/2 when co-treated with Epitalon. Furthermore, the incubation of THP-1 macrophages with Epitalon did not induce the phosphorylation of latent cytoplasmic transducers, as evidenced by the lack of significant increase in activated molecules detected by anti-phospho STAT3 antibodies. Furthermore, the Epitalon time-course treatment appeared to further diminish the phosphorylation level of phospho-STAT3 [46]. In another study, it was shown that Epitalon had an effect on macrophages depending on the age of mice from which the cells were collected. Three doses (0.0025 ng/mL, 0.025 ng/mL, and 0.25 ng/mL) were investigated. Macrophages from young mice decreased the production of lymphocyte-activating factors under the influence of Epitalon by a statistically important value. Activity in old mice macrophages was not influenced in a statistically important manner [47,48].

A recent study mentioned an interesting use of Epitalon. Yue et al. connected the antioxidant [11,49] and protective [50] properties of Epitalon with the possible use of this compound as an in vitro oocyte-protecting agent. The existence of intracellular reactive oxygen species (ROS), the frequency of spindle abnormalities, the location of cortical granules, and mitochondrial activity were examined. The ROS level was examined following 24 h of culture with various doses of Epitalon. Concentrations of 0.05 mM and 0.1 mM markedly reduced the ROS levels. It is noteworthy that elevated concentrations of Epitalon (1 mM and 2 mM) did not substantially reduce ROS levels. Epitalon has also shown a reduction in oocyte fragmentation. The highest results were again seen at the lowest concentration of Epitalon—0.1 mM. The localization of cortical granules, mitochondrial membrane potential, and mtDNA copy number were also influenced by Epitalon. Epitalon consistently demonstrated activity that can be characterized as geroprotective. The final criterion examined in this study was DNA damage and apoptosis in oocytes. The fluorescence intensity of γH2AX was utilized as an indication of DNA damage. DNA damage was reduced in comparison to the aged group following the addition of Epitalon during culture. Epitalon in vitro decreased the incidence of oocyte apoptosis. All data obtained by the authors indicated that Epitalon is a viable choice for oocyte protection in in vitro cultures [51]. Identical qualities were determined to be beneficial in the culture of human gingival mesenchymal and periodontal ligament stem cells. Cells subsequent to the 25th passage were administered Epitalon. RT-PCR measurements revealed that the expressions of the senescence markers p16 and p21 were reduced by 1.56 and 2.22 times, respectively, compared to the control (*p* < 0.01) in periodontal ligament stem cells, and by 1.92 and 2.44 times in gingival mesenchymal stem cells [52].

Research conducted on isolated primary rat fibroblasts and organotypic cultures of rat skin explants has revealed that Epitalon possesses anti-aging activities in skin tissue as well. Molecular evidence was established by quantifying the expression levels of Ki-67, CD98hc, caspase-3, and MMP-9 in fibroblasts treated with AEDG [53]. Gutop et al. also noted increased expressions of genes encoding SOD-1, NQO1, and catalase in human cells undergoing Epitalon exposure. They also emphasized the potential for Epitalon to directly attach to the promoters that govern the expression of these enzymes—Keap1/Nrf2 [54]. Experiments conducted on complete organotrophic cultures support this hypothesis [55].

Recently, the neuroprotective effects of Epitalon were examined in vitro. AEDG was reported to reduce DNA damage in neurons generated from fibroblasts, indicated by a drop in 8-hydroxydeoxyguanosine levels,. Furthermore, the quantity of primary and terminal dendrites escalated following Epitalon administration. The total dendritic length and the quantity of junctions were also augmented. Other peptides examined in the same study demonstrated marginally superior outcomes [50].

A study published in 2025 by Ullah et al. demonstrated novel features of Epitalon in relation to telomerase. Epitalon was demonstrated to stimulate telomerase activity in bovine cumulus cells and cumulus–oocyte complexes. The impact of Epitalon on mitochondrial health was examined using JC-1 staining. Epitalon enhanced mitochondrial health and reduced intracellular reactive oxygen levels. Subsequent analysis revealed that the mRNA expressions of PGC-1α, Sirt-1, tFAM, and BCL2 were markedly elevated following Epitalon administration. These data were utilized in the assessment of whether Epitalon is an appropriate supplement for media used in oocyte maturation. The maturation process was discovered to be accelerated by improving the mitochondrial health of oocytes. Moreover, Epitalon therapy of post-thawed bovine embryos markedly improved blastocyst expansion and hatching rates, Table 1.

### 2.2. Drosophila melanogaster

*D. melanogaster* has been identified as a valuable model for assessing lipid peroxidation levels. Highly inbred strains were selected by the reduced mating potential of males. Epitalon was added into the nutritional medium at the larval stage at a concentration of 0.00001% (*w*/*w*). The results indicated that adult flies had reduced amounts of conjugated hydroperoxides following Epitalon treatment during the larval stage. An identical outcome was also seen for the concentrations of Schiff’s bases. The results varied by gender, although in both instances, the observed alterations following Epitalon administration indicated reduced concentrations. These results demonstrate that Epitalon undoubtedly possesses antioxidant properties. Furthermore, the authors indicated that, upon comparison with the literature, the effective concentration was sufficiently low to suggest a signaling role for Epitalon in the cascades of the cellular antioxidant system [57]. The second article by the same authors from the same year additionally investigated catalase activity. Again, Epitalon had antioxidating effects. Additionally, Epitalon was compared with its natural occurring progenitor, Epithalamin. The results of the comparative analysis in this study revealed that Epitalon is a better antioxidant than Epithalamin. It is important to note that Epithalamin was administered at 1000-fold higher doses [58]. A similar experiment focused on measures of reactive oxygen levels in flies. Epitalon was compared with Vilon. Both peptides inhibited reactive oxygen formation in mitochondria and cytosol [59].

Epitalon has an influence on the lifespan of *D. Melanogaster.* A wild strain, Cantos-S., of *D. Melanogaster* was used as a model in an experiment, where early developmental stages were treated with Epitalon at different concentrations. Overall, Epitalon increased the lifespan of imago *D. Melanogaster* by up to 16%. This effect was observed for both genders. Survival curves additionally confirmed that Epitalon mainly influenced mature and old imago flies. Khavinson et al. stated that these results confirmed the lack of genotoxic effects potentially conducted by Epitalon [60]. What was special about Epitalon were the doses which induced the effect. To achieve comparable results for a medium life span extension for *D. Melanogaster,* melatonin has to be implemented at 16,000-fold higher concentrations [61].

### 2.3. Mice

One of the *main* biological models in Epitalon research is mice. The influence of Epitalon on the occurrence of chromosomal abnormalities was examined in comparison to another epiphyseal hormone, Melatonin. The study included the following three strains of mice: SAMP-1, SAMR-1, and SHR. Chromosomal abnormalities were quantified in bone marrow following 10 months of treatment with Epitalon or Melatonin (only SHR). SAMP-1 mice exhibited a greater frequency of chromosomal abnormalities in bone marrow cells relative to normally aging SAMR-1 mice and long-lived SHR mice of an equivalent age. In all instances, the administration of Epitalon reduced this parameter. The impact of Epitalon was greater than that of melatonin treatment in SHR mice [62]. SHR mice were used as a model in a study that examined biomarkers of aging. Epitalon was found to have no effect on food intake, body weight, or average lifespan in this specific strain of mice. Nonetheless, similarly to the previously referenced article, bone marrow chromosomal aberrations were diminished by 17.1% (*p* < 0.05). This study found no effect of Epitalon on overall tumor incidence [63].

Epitalon decreases chromosomal instability [64]. Chromosomal aberrations are directly connected to DNA defects, which are often responsible for carcinogenesis. This parameter was investigated in a long-term study where Epitalon was administered to CBA mice in 10-fold lower doses (0.1 µg/mouse). Epitalon reduced the incidence of overall tumor formation (*p* < 0.05) and their multiplicity [65]. Reduced carcinogenesis was also confirmed in FVB/N female mice transfected with the HER-2/neu breast cancer gene. The total number of breast adenocarcinomas was found to be lower in comparison to a control group (*p* < 0.05) [66,67]. Further investigation during following studies confirmed a 3.7-fold lower HER-2/neu mRNA expression in FVB/N female mice compared to controls [68]. Epitalon decreased tumor size in specific tissues [69,70]. It was confirmed that FVB/N HER-2/neu mice treated with Epitalon had mammary tumors of a smaller size [67]. These results are in line with Kossoy’s et al. experiment, which was performed on another mice strain, C3H/He. Again, the antitumor effect of Epitalon was confirmed in a 6.5-month-long study by comparing the tumor incidence, localization, and type compared to a control group treated with saline subcutaneously [71].

Semenchenko et al. proposed a semi-parametric model of heterogeneous mortality (frailty model) for the analysis of experimental data. In their experiment on HER-2/neu transgenic mice, many factors were investigated, and one of them was Epitalon administered in two treatment schemes. Depending on the dosage, Epitalon was observed to either reduce the maximum lifespan of mice—due to debilitation and the aggregation of frail individuals in the population—or significantly enhance survival rates in transgenic mice—attributable to adaptability, increased average robustness, and heterogeneity [72].

The antimutagenic effects of Epitalon were also examined in relation to the hair color of the mice in the study. Mylnikov et al. employed albino and grey mice to examine the potential color dependence of results in the following two assays assessing antimutagenic activity: the incidence of abnormalities in sperm heads and the occurrence of micronuclei in peripheral blood erythrocytes. Epitalon demonstrated significant antimutagenic action in both experiments using gray mice, and in both tests with albino mice, it exhibited enhanced antimutagenic activity. Due to the atypical nature of these data, the authors offered various complementary explanations. Furthermore, they emphasized the possible intricacy of this issue [73].

The specific effects of Epitalon on gene expression in the heart were studied in vivo [74]. Overall, 15,247 genes were investigated. Epitalon activated the expressions of 194 genes up to 6.61 times in some cases. Inhibition occurred on the expression of 48 genes by up to 2.71 times in the most inhibited case. The authors provided specific information about the genes affected. These data showed consistency with the previously stated biological properties of Epitalon, where this compound was found to inhibit the development of spontaneous tumors [74]. Next, 16,897 transcripts were studied in mice brains after Epitalon administration. Mice were treated with 10 times lower doses compared to the experiment examining the heart. The effects on 53 gene expressions were presented. This study revealed major differences in mice brain gene expression after Epitalon treatment compared to Melatonin treatment. The effects of Epitalon were mostly connected with genes which are responsible for nucleic acid transport and synthesis, apoptosis, and cell cycle regulation [75].

The influence of Epitalon on mice body weight dynamics and many other parameters was investigated in a long-term study on the CBA mice strain by V.N. Anisimov et al. Epitalon did not significantly increase or decrease body weight in the 21-month period of the study, compared to controls. Also, no significant changes in food consumption during the study were reported. Within the same study, physical activity was also investigated. It was shown that, at the start of the study (the first 3 months of Epitalon administration), the physical activity of mice decreased significantly (*p* < 0.001). However, this parameter changed later in time and did not vary significantly from the control group at the end. Muscular strength and physical fatigability were not affected by Epitalon throughout the study period. Also, no statistically significant changes were reported in age-related estrus functionality after long-term Epitalon administration. One of the most interesting results within this study was in line with the thesis that Epitalon shows geroprotective activity. It was shown (*p* < 0.01) that the number of mice treated with Epitalon that reached the age of 23 months surpassed the control group 4.0-fold. The oldest mice in the saline control group died at 24 months old and the oldest mice in the Epitalon treated group lived for 34 months [65]. This aligns with findings from studies examining the lifespans of a different strains of mice [76], for example, FVB/N female mice transfected with HER-2/neu, which likewise validated the extended lifespan of Epitalon-treated mice (*p* < 0.05) [66], or the study in which it was found that Epitalon administration extended the lifespan of the last 10% of survivors of SHR mice by 13.3% [63]. At the end of this investigation, therapy with Epitalon significantly suppressed the generation of free radicals in brain tissue. This peptide formulation also significantly prevented lipid peroxidation in the brains and livers of CBA mice, Table 2 [65].

### 2.4. Rats

The majority of articles mentioning Epitalon use rats as model animals for in vivo experiments. Similarly to experiments performed on mice, many strains were used and widely investigated.

Epitalon was found to be a gastric endocrine cell behavior-modifying agent [77,78]. In a study performed on Wistar rats, before Epitalon subcutaneous injection, rats were pinealectomized. Epitalon was administered for 30 or 42 days. Serotonin-producing enterochromaffin cells, gastrin-producing cells, and somatostatin-producing cells were counted. The number of above-mentioned cells in pinealectomized rats without Epitalon administration did vary significantly compared to controls. Epitalon did, to a certain degree, decrease the effect of pinealectomy on gastric cells. For example, the ratio between gastrin- and somatostatin-producing cells stayed at the same level as those in the control group after 30 days of Epitalon administration. Forty-two days of subcutaneous Epitalon administration fully recovered the pinealectomy-induced changes in gastric endocrine cells to the levels of the control group. This experiment led V.K. Khavinson et al. to the conclusion that Epitalon has a direct impact on gastric endocrine cells [78]. Another study examined the characteristics of the rat duodenum following γ-irradiation. Intraperitoneally administered Epitalon inhibited proliferation and exhibited no additional post-γ-irradiation activity. The authors stated that such activity of Epitalon could be beneficial in antitumor treatments [79]. This statement stays in line with the results obtained by V.N. Anisimov et al., where Epitenon significantly decreased the amount of cancer incidence in a 1,2-dimethylhydrazine-induced colon carcinogenesis model [80]. In 2003, the anti-cancer activity of Epitalon was confirmed in different developmental stages of colon cancer. Epitalon is especially effective when applied before, during, and after cancer occurrence [81].

Epitalon also exerted a regulatory influence on pepsin activity during aging in unfavorable light conditions of the Karelia Republic. Nevertheless, it was demonstrated that Epitalon did not significantly affect overall proteolytic activity. Research demonstrated that pepsin activity and overall proteolytic activity remained unchanged by Epitalon under 12 h light/dark cycles or constant light [82].

The impact of pinealectomy on the spleen was similarly examined. Subcutaneous injections of Epitalon or Epithalamin were administered to pinealectomized rats. Similar to the previous experiment involving intestinal cells, Epitalon and Epithalamin only partially mitigated the impact of pinealectomy on spleen cells. It was confirmed that Epitalon and Epithalamin significantly affect splenic functionality and morphology. The results for groups administered with Epitalon and Epithalamin exhibited statistically significant differences compared to the control group and rats without splenic activity adjustment [10].

As previously mentioned, Epitalon shows geroprotective activity [83]. This activity is usually connected with antitumor activity. This was also confirmed in an experiment performed on male LIO rats, where tumor incidence depending on Epitalon administration and light exposure was investigated. Epitalon did significantly lower tumor incidence in all three light exposure schemes (*p* < 0.02). However, Epitalon did not prolong the mean lifespan in all groups [84]. Tumor incidence dependence on Epitalon administration and light exposure was also investigated in female LIO rats. In this experiment, rats exposed to light natural for northwestern Russia and constant light showed a prolongation of their maximum life span. The results also presented that spontaneous tumor incidence for female rats was lowered only in natural northwestern Russia light conditions [85].

Another anti-aging characteristic of Epitalon, previously emphasized, is anti-free-radical oxidation inhibition. Based on the lipid peroxidation rates in the brain and serum of Wistar rats, this property of AEDG was confirmed in vivo in rats by L.S. Kozina [1]. The next anti-aging related property of Epitalon is the inhibition of apoptosis. To validate this property in rats, Wistar rats were subjected to γ-irradiation, and the apoptosis rate of their splenic lymphocytes was assessed. On day 2 post-irritation, Epitalon was delivered intraperitoneally with physiological saline to one group for the subsequent 5 days. Epitalon reduced the apoptosis of splenic cells by 2.12-fold (*p* < 0.05) compared to the control group that received only saline injections [86]. A separate investigation utilizing γ-irradiation to examine the protective qualities of Epitalon in rats yielded noteworthy results. Microscopic examination revealed that Epitalon partially preserved the pineal gland from structural changes and facilitated its recovery, particularly through the partial restoration of the mitochondrial ultrastructure. This occurrence might be seen as an additional instance of the tropic activity of Epitalon on the pineal gland [87].

The activity of the pineal gland is influenced by Epitalon. In a study examining stress-exposed rats, the tetrapeptide was administered intranasally. The occurrence of C-Fos protein, which is a neuron activation marker [88], was measured. Epitalon slightly, although statistically significantly, elevated the levels of C-Fos protein in the pineal gland, exclusively in rats subjected to stress [89]. An elevated expression of C-Fos protein was also seen in all hypothalamic regions in both stress-adapted and stressed rats [90].

**Figure 3:**
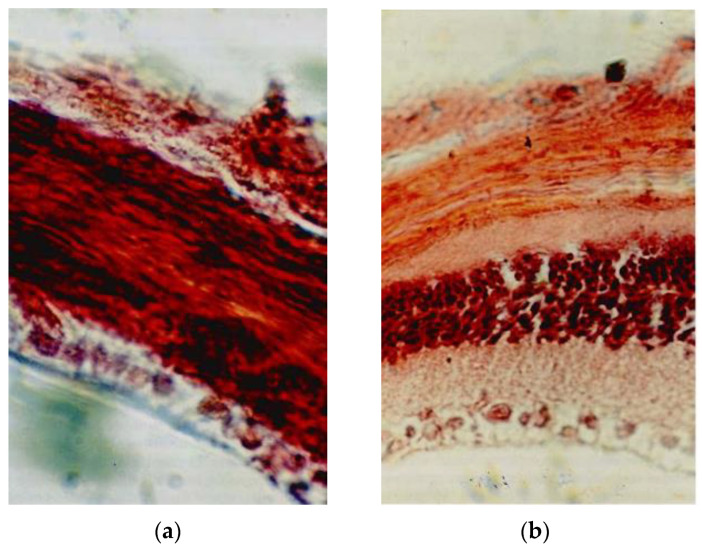
Morphologic pictures of retina in Campell rats on the 41st day in the control group (**a**) and in Epitalon injected parabulbarly treatment group (**b**). Hematoxylin–cosin staining, ×700 magnification. Reprinted with permission from [20], licensed under CC BY-NC-ND 4.0.

Based on the similarities of Epitalon to Retinalamine [8], an interesting branch of studies performed on rats emerged. Campbell rats were injected parabulbarly with Epitalon for 72 days starting at birth. Retina tissue was morphologically investigated. On day 41, the complete destruction of all retinal layers was found in rats which had not been treated with Epitalon, while in the experimental group, all retinal layers were preserved. Electroretinogram measurements indicated that Epitalon prolonged the functional activity of the retina by 43.9% [18]. Superior outcomes (two-fold extension of functional activity) were achieved when female Campbell rats received intraperitoneal injections of Epitalon commencing three weeks prior to gestation. The results confirmed that parabulbar injections are unnecessary for the desired effect and that Epitalon is more effective when administered during pregnancy and throughout life [19]. Considering these results, the authors attested that Epitalon is highly efficient in some forms of retinal degeneration [18]. Another study where five times lower doses were used also showed significant improvement in the course of *Retinitis pigmentosa*, Figure 3 [20].

It was also shown that Epitalon has an effect when administered orally. In total, 100 µg of Epitalon was given orally to rats for 1 month. Epitalon increased passive glucose transport in the medial and distal segments of the small intestine 2.2-fold (*p* < 0.05) and by 40%, respectively. Active glucose transport was also highly affected by Epitalon, increasing its value in the proximal and medial regions by 6- and 8-fold, respectively [13]. Epitalon has been found to affect enzymatic functions [91], including those of the digestive system [92,93]. One experiment confirmed that this activity is age-related, as higher results were observed in older Wistar rats (11 months old) compared to younger ones (3 months old). Epitalon significantly (*p* < 0.05) enhanced invertase (EC 3.2.1.48), maltase (EC 3.2.1.20), and glycyl L-leucine dipeptidase (EC 3.4.13.2) activities in a regioselective manner [92].

Epitalon demonstrates central effects and functions as an immunomodulator [94,95]. This was demonstrated using intramuscular and intranasal injections of Epitalon. Research demonstrated that, after 24 h for intramuscular administration and 1.5 h for nasal application, Epitalon enhanced IL-2 mRNA synthesis rates in the lateral hypothalamus area, anterior hypothalamic fields, dorsomedial, ventromedial, and paraventricular hypothalamic nuclei [96]. In a similarly conducted experiment on Wistar rats, which additionally involved mild stress and utilized smaller doses of Epitalon, the quantity of IL-2-positive cells was examined. IL-2 positive cells in the anterior hypothalamic nucleus, paraventricular nucleus, and supraoptic nucleus diminished at 24 h following the intramuscular administration of Epitalon under mild stress conditions. Intranasal treatment resulted in a reduced quantity of IL-2 positive cells in the lateral hypothalamic area, while the anterior hypothalamic nucleus remained unaffected [97]. A regioselective IL-2 expression decrease was also confirmed when Epitalon was administered intranasally [90].

Epitalon also influences age-related alterations in the estrous cycle. The administration of Epitalon significantly prolonged the cycle’s duration, particularly in 17-month-old female rats. The study demonstrated that Epitalon was effective in an experimental model of premature reproductive aging induced by 1,2-dimethylhydrazine administration. This drug reinstated the normal daily dynamics of neurotransmitters in hypothalamic regions important for the production and release of gonadotropin-releasing hormone. It was suggested that Epitalon is particularly effective in mitigating adverse ecological impacts on the reproductive functions of young, adult, and aging female creatures when provided alongside another chemical derived from the pineal gland—Melatonin [98]. This activity of Epitalon could be connected with the ability of Epitalon to regulate the dopamine level in arcuate nuclei post-1,2-dimethylhydrazine injection [99].

The intranasal administration of Epitalon can influence the central nervous system. Experimental evidence has demonstrated that spontaneous neuronal activity in the parietal and frontal neocortex regions of Wistar rats was enhanced, as shown by an increased absolute neuronal discharge frequency. No substantial increase in neuronal activity was observed during the first three minutes after administration. The most substantial rise was observed between 5 and 7 min following Epitalon injection. A microinjection of Epitalon into the intracellular medium of the neocortex demonstrated a similar increase in activity, with the maximum activity peaks recorded from 10 to 20 s post-injection. The mechanism of this phenomena remains undisclosed [100].

Epitalon is not nephrotoxic for rats [101]. Due to Epitalon’s structural similarity to other synthetic bioactive kidney peptides, T-35 (Glu-Asp-Leu) and T-31 (Ala-Glu-Asp), it was investigated as a potential treatment in rhabdomyolysis. In a glycerol intramuscularly injected model of acute kidney failure, Epitalon, with statistical significance compared to rats with acute kidney failure, increased diuresis (mL) and decreased protein excretion (mg/h). Additionally, Epitalon increased the activity of catalase and glutathione peroxidase. These results led I.I. Zamorskii et al. to a statement that Epitalon can be beneficial in rhabdomyolytic kidney failure treatments [102]. The same author demonstrated a year later in another article that Epitalon injections mitigate the adverse effects of acute kidney failure generated by cisplatin on renal functioning [103]. Epitalon’s nephroprotective properties were also proven in old rats without induced nephropathologies, Table 3 [101].

### 2.5. Rhesus Monkey

**Figure 4:**
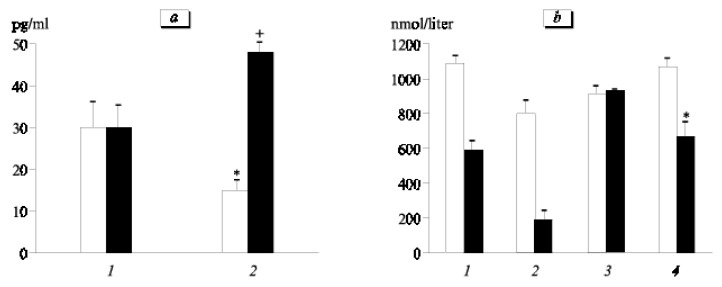
Impact of Epitalon on melatonin production (21:00, **a**) and cortisol levels (09:00 and 21:00, **b**) in monkeys of differing ages. (**a**) Light bars: control (placebo); dark bars: Epitalon. Ages: 6–8 years (1) and 20–26 years (2). * *p* < 0.01 in comparison to control young subjects; + *p* < 0.001 in comparison to control old subjects. (**b**) Light bars: 9.00; dark bars: 21.00. Ages: 6–8 years (1 and 2) and 20–26 years (3 and 4). Control (1 and 3) and Epitalon (2 and 4). * *p* < 0.05 compared to control group of older animals at 21:00. Reproduced with permission from Springer Nature [104].

Multiple studies on Rhesus monkeys (Macaca mulatta), researching the effect of Epitalon administration, have been conducted. In the first experiment, N.D. Goncharova et al. measured the cortisol and melatonin levels in old monkeys (average age 22.8 ± 1 years) after an intramuscular injection of Epitalon. Epitalon showed differences depending on the time of the experiment and the age of the monkeys (as a control group, young monkeys were used). Epitalon mostly influenced old monkeys by stimulating melatonin synthesis and normalizing cortisol blood concentrations depending on the time of day, Figure 4 [104,105].

Three years later, in 2004, other results of experiments involving Macaca mulatta were published. This time, the impact of Epitalon on plasma glucose concentration was investigated. Young and old monkeys were treated with Epitalon intramuscularly, and their blood glucose concentrations were measured. Before treatment with Epitalon, old monkeys had a glucose response area with significantly higher values compared to young monkeys (479.6 ± 38.0 mM/min and 294.9 ± 9.3 mM/min, respectively). Epitalon administration lowered the glucose response area value in old monkeys to 388.9 ± 43.6 mM/min and increased the value for young monkeys to 343.3 ± 48.2 mM/min. After one month without Epitalon administration, these values returned to closer to their initial values. The authors of this study stated that this effect could be correlated with Epitalon’s ability to increase melatonin levels, which is thought to influence the glucose sensitivity of cells of Langerhans islets [106]. A separate study conducted similarly confirmed that melatonin levels are correlated with an improved glucose tolerance with prolonged dosing (10 days). These results were observed exclusively in aged monkeys (20–27 years). Young monkeys remained largely unimpacted [107].

### 2.6. Clinical Trials

Previous promising results on Campbell rats, where Epitalon was found to be a suitable drug for *Retinitis pigmentosa* [18,19,20], led to a clinical trial. The trial was performed at the St. Petersburg Institute of Bioregulation and Gerontology. Overall, 162 patients with *Retinitis pigmentosa*, aged from 18 to 72 years, were examined. Each patient received 5.0 µg of Epitalon per eye, injected parabulbarly for 10 consecutive days. The control group was treated with conventional methods for 2002 (antisclerotic agents, vasodilators, and angioprotectors). None of the Epitalon-treated patients reported side effects. The amplitude activity of both the first and second neurons rose significantly. The visual acuity of treated patients increased by 0.15–0.20 on average. The peripheral borders of the visual field were extended in all patients. A total of 64.8% of patients had their total visual field border broadened by 90–120 degrees. Absolute scotomas reduced in size and some of them even disappeared. These results evidence that Epitalon treatment is suitable for *Retinitis pigmentosa* treatment [20].

A separate study conducted on 75 women examined the ability of Epitalon to regulate and preserve the circadian rhythm. Epitalon was delivered sublingually for a duration of 20 days at a dose of 0.5 mg/day. The production of melatonin in the epiphysis and the expression of human circadian genes were examined. The research comprised placebo and control cohorts. Melatonin synthesis in the epiphysis was assessed by quantifying the excretion of 6-sulfatoxymelatonin in urine. Epitalon was observed to enhance this parameter by 1.6 times relative to the placebo group. The circadian genes examined were the Clock and Cry2 genes in leukocytes and the Csnk1e gene in lymphocytes. Clock gene expression was reduced by a factor of 1.8 relative to the placebo. Following the administration of Epitalon, the Cry2 expression in leukocytes was doubled (*p* < 0.05) compared to pre-treatment levels, while the Csnk1e expression in blood cells decreased by 2.1 times (*p* < 0.05). The scientists concluded that Epitalon’s geroprotective activity is attributed to its capacity to restore epiphyseal melatonin production through the modulation of human clock gene expression [108].

### 2.7. Other Biological Models and Experiments

Epitalon was also administered to Chinchilla rabbits. The objective of the study was to examine Epitalon binding in maternal and fetal tissues in both healthy conditions and instances of placental insufficiency by quantifying the fluorescence of dansyl-labeled Epitalon. Epitalon was administered subcutaneously one hour prior to sacrifice via air embolism. The experiments revealed the swift integration of Epitalon into metabolic processes across nearly all maternal and fetal tissues during normal pregnancy and in cases complicated by placental insufficiency [109].

Epitalon’s inhibitory effect on human enkephalinase was observed. Inhibition was dose-dependent. The IC_50_ for Epitalon was 500 μM, and was not the lowest value within the study. The reliability of these results was confirmed by the implementation of known enkephalinase inhibitors in the experiment, which gave the above results. Additionally, Epitalon did not affect the binding of a tritium-labeled synthetic analog of an endogenous opioid peptide to the rat brain membranes, indicating that these peptides did not interact with μ- or δ opioid receptors [110].

**Figure 5:**
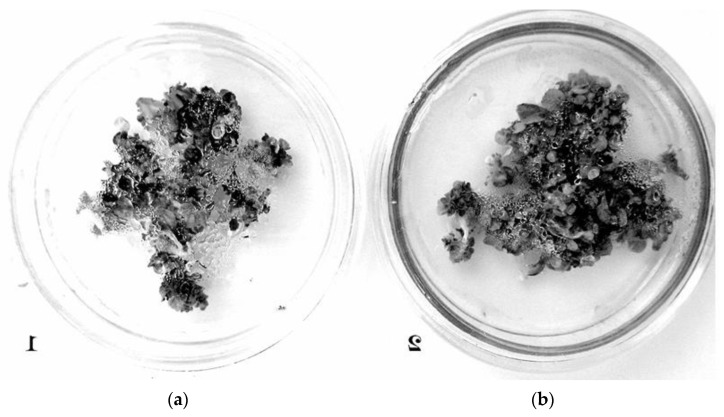
Influence of Epitalon at concentration of 10^−8^ M on growth of tobacco callus cultures. Control (**a**) and Epitalon-treated culture (**b**). Reproduced with permission from Springer Nature [111].

A novel approach to studying Epitalon is to look into its impact on plants. In a specific investigation, Epitalon was delivered to *Nicotiana tabacum L.* by culturing its calluses on agar media infused with Epitalon. The initial results indicated a volumetric increase in callus tissue mass cultivated in media containing Epitalon. However, concentrations exceeding 10^−6^ M inhibited the growth rate. Secondly, the expression levels of CLE (CLE1–CLE6), plant growth regulatory factors (GRF1–GRF4), and KNOX (KNAT1-KNAT3, KNAT6, LET6, and LET12) groups were quantified using real-time PCR. This study was the first to demonstrate that Epitalon influences the expression levels of several genes within this category. However, this topic remains mostly unexamined, Figure 5 [111].

*Nicotiana tabacum* L. also serves as a valuable model in the examination of the epigenetic effects of Epitalon. Epitalon, as previously noted, enhanced the proliferation of calluses at low doses (10^−7^ M). Subsequent analysis verified that Epitalon exhibits epigenetic action by specifically binding to the CAG region of DNA, which serves as a methylation target for plant cytosine methyltransferases. Such activity of this tetrapeptide may activate or silence the expressions of certain genes [112].

## 3. Knowledge About Epitalon Based on Physico-Chemical Studies

Apart from in vitro and in vivo experiments, Epitalon has also been studied using in silico approaches. Epitalon’s molecular properties in aqueous solution were calculated at the molecular mechanics level [29,113]. Molecular dynamics simulations of Epitalon in zwitter-ionic form were conducted using an AMBER force field, with a 37.5 Å × 22.5 Å × 13.5 Å rectangular box containing 347 solvent molecules. A TIP3P model was used to represent water molecules during calculations. The simulation lasted 1500 ps and the temperature of the calculated system was set to 300 K. The conformer structure with the lowest energy and 120 other conformers were obtained. It was found that Epitalon forms two intramolecular salt bridges which stabilize its structure. Both of these salt bridges are formed between the nitrogen atom of alanine and non-peptide-bond-forming carboxylic groups of glutamic acid and aspartic acid. Additionally, an intramolecular hydrogen bond was recognized. Rogachevskii et al. stated that these intramolecular bonds highly decrease the conformational freedom of Epitalon [114].

**Figure 6:**
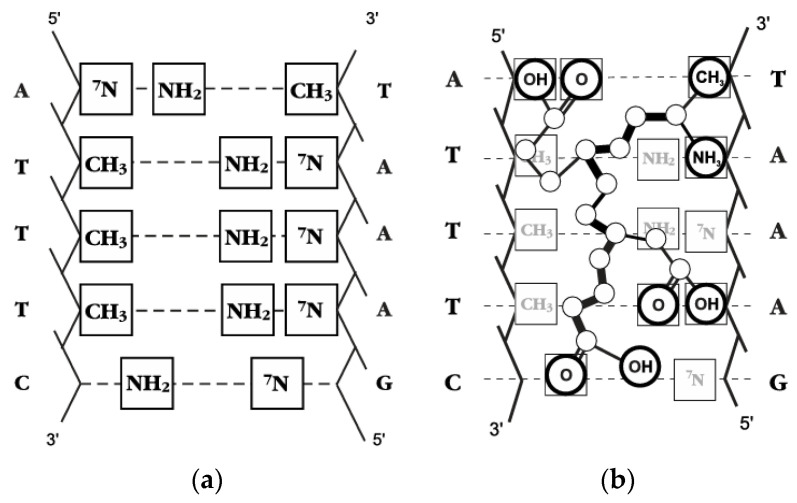
Sequence of nucleotide pairs in the DNA double helix with functional groups that are complementary to the functional groups of Epitalon (**a**) and the “interactions graph” (**b**) of Epitalon and ATTTC base sequence. Reprinted with permission from [115], licensed under CC BY-NC-ND 4.0.

Epitalon was also studied in silico in 2003 by investigating its interactions with DNA fragments. Epitalon was found to “unbound” to a β-like structure and interact with a specific ATTTG DNA sequence by hydrophobic interactions and hydrogen bonds. Hydrophobic forces were observed between the side chains of the peptide and the methyl groups of thymine. However, this article provided no precise data about the structure of such a complex [113]. The topic of DNA binding was further discussed in 2004. Again, Epitalon was found to interact with a specific DNA sequence—this time, ATTTC. A 3D model of this complex was not provided by the authors, but a more specific “interactions graph” was presented (Figure 6). The exact consequence of the binding of this molecule to this particular base sequence is not known, however, Khavinson V.K. et al. correlated this characteristic of Epitalon with its specific biological action, especially because this specific sequence is present multiple times in the promoter part of the telomerase gene [115].

Investigations into the thermodynamics of DNA have demonstrated that incremental temperature elevations lead to a phase shift in the “helix–coil”, also known as DNA melting [116]. This phase transition causes the double helix to dissociate into two single strands, manifesting as random coils. A comparable reversible helix–coil transformation transpires at ambient temperature, without thermal application, when DNA is subjected to acidic and alkaline solutions. This random coil transition is associated with an increase in absorbance at 260 nm, referred to as the hyperchromic effect [117]. A. Solovyev et al. discovered that the presence of Epitalon reduced the melting temperature of double-stranded DNA by as much as 41 °C at an ionic strength equivalent to a 0.1 M NaCl concentration [118].

The molecular docking of Epitalon to other molecular targets was also performed. In 2013, Fedoreyeva et al., based on an experiment that revealed interactions between Epitalon molecules and labeled histones, proposed that the epigenetic effect of this molecule is not only related to interactions with DNA [119]. Seven years later, these results led to an Epitalon/histone complex Molecular Dynamics (MD) analysis, based on Molecular Mechanics (MMs), using an Amber12EHT force field. Overall, 50 docking results with 6 different histone proteins were investigated. The core histones H2b, H3, and H4 did not reveal any binding sites suitable for Epitalon. It was found that Epitalon most likely forms complexes with histones H1/6 (binding energy: −64.51 kcal/mol) and H1/3 (binding energy: −56.49 kcal/mol). This is especially important, because these proteins interact with DNA helixes through these sites. This is consistent with the thesis stated before [40]. A subsequent molecular docking experiment was conducted by docking Epitalon to the transporter proteins LAT1, LAT2, PEPT1, and PEPT2. Values for the scoring functions of Epitalon indicating strong binding, represented by the ICM-Score, were acquired (Table 4).

Epitalon has a binding strength comparable to that of established PEPT1 inhibitors. The authors of this work indicated that the anti-cancer capabilities of Epitalon, previously demonstrated in in vitro and in vivo experiments, may be associated with the blockage of the amino acid transporters LAT1, LAT2, and PEPT1 [120].

The possibility of Epitalon forming complexes with lysine dendrimers was also investigated in silico using MD. The time taken for the evolution of a system containing 16 molecules of Epitalon with second- and third-generation lysine dendrimers in a water environment was investigated. Simulations lasting 160 ns showed the complete absorption of all peptide molecules on the dendrimer. Complexes between 16 Epitalon molecules and second-generation dendrimers were formed after 20 ns. The radial distribution functions of atoms were computed. The results indicated that dendrimer atoms mainly resided within the complex, whereas the majority of peptide atoms were located on its surface. The primary interactions between Epitalon molecules and dendrimers transpired between the positively charged NH_3_^+^ groups of the dendrimer and the negatively charged COO^−^ groups of glutamic acid and asparagine in Epitalon peptides. Consequently, the findings indicated that the binding strength between these two molecules can be altered through pH manipulation, which is, indeed, accurate. The authors of this paper asserted that such complexes may prove beneficial in future oral methods of administration for Epitalon [121]. The latest work on this subject further examined the complex formation between Epitalon and lysine dendrigrafts, as well as K2R dendrimers. Complexes were produced rapidly (within 10 ns). The Epitalon–dendrimer complex was determined to possess a minimum atomic density. These macromolecular complexes represent a possible solution for modern medication delivery methods [122].

Epitalon was briefly mentioned in an article that focused on potential *Staphylococcus aureus* shikimate dehydrogenase (SaSDH) inhibitors. Epitalon, in the form of trifluoroacetate salt, and 11 other compounds were selected from the MCE Bioactive Compound Library and implemented in molecular docking calculations using the Glide module in Schrödinger Maestro 11.4 to assess SaSDH. Epitalon showed no major inhibitory effect on SaSDH. Its Docking Score was −10.154, its IC_50_ (μM) was 1097.1, and its minimal inhibitory concentration was found to be above 100 μg/mL. No further investigation into Epitalon was performed within that study [123].

Its influence on oxidation reactions was also confirmed. Epitalon was tested in an experiment were methyl oleate Fe^2+^-induced oxidation rate was measured. Epitalon showed no major difference in oxidation inhibition depending on the applied concentration [12].

An analytical approach to Epitalon was presented by Vanhee et al., where products available on the market, labeled as Epitalon-containing, were investigated. Liquid chromatography–tandem mass spectrometry (LC-MS/MS) was used to investigate the reference sample and the suspected pharmaceuticals. Full MS spectra of Epitalon were recorded. During the investigation, the risk of counterfeit by the Glu-Ala-Asp-Gly (EADG) tetrapeptide was considered. Absolute distinction was obtained by heat treatment of the suspected peptide [124] The fact that N-terminal glutamic acid can cyclize to form pyroglutamate [125] and lower the mass as a result was used by the authors. This confirmed the presence of Epitalon in products available on the market. To sum up, it was confirmed that LC-MS/MS is an appropriate method for Epitalon detection in pharmaceutical products [124].

Two years later, in 2017, Epitalon’s presence was, for the first time, reported directly in pineal gland polypeptide complex solution. LC-MS was, again, crucial for obtaining these results [9].

## 4. Design of the Study

**Figure 7:**
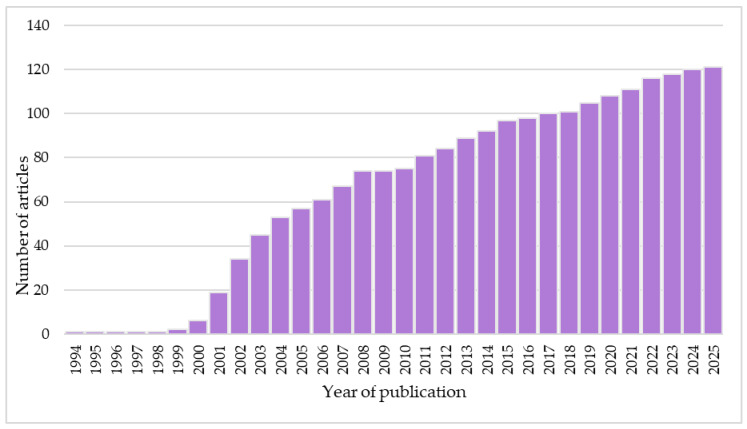
Number of search results for articles describing Epitalon. The graph shows the cumulative number of records in Scopus in the period of 1994–2025. Each column shows the cumulative number of articles in the given year and all years before. For example, the column entitled ‘2015’ depicts the number of articles published in the period of 1994–2015, including 2015.

Two independent evaluators (S.A. and Ł.S.) were appointed to select the articles for this review. The examiners conducted an extensive literature review on Epitalon in the Scopus and PubChem databases. The following keywords were incorporated among the search terms: “Epitalon”, “Epithtalone”, “Epithalon”, “Epitalone”, “AEDG”, “Ala-Glu-Asp-Gly”. This review sought to compile and examine the studies on Epitalon, regardless of the method and type of study. Consequently, the inclusion criteria for this review were either the application of Epitalon or studies devoted to the analysis of the physico-chemical properties of this peptide. The exclusion criteria for this study encompassed the exclusive use of other peptides or the sole application of the mixture of amino acids that Epitalon is composed of. Upon completing the inclusion and exclusion procedures, any disputes were resolved through consensus among the reviewers. A comprehensive examination of the included papers was undertaken to ascertain any other pertinent research that could be incorporated into the evaluation (Figure 7).

## 5. Future Perspectives

As shown in this review, Epitalon exhibits significant and multidirectional pharmacological activity. However, in the analyzed works, information regarding critical issues about this peptide’s safety is missing. Before Epitalon’s approval as a new API, additional studies on its potential short- and long-term toxicity are essential [56]. These future studies should also include aspects such as genotoxic activity, carcinogenic potential, and food–drug and drug–drug interactions.

Furthermore, short peptides such as Epitalon are typically unstable and degrade rapidly in vivo. Chemical modifications like acetylation and amidation can sometimes be employed to enhance the stability of such peptides. However, to the best of our knowledge, in the case of Epitalon, this idea has not been evaluated yet.

Also, due to the presence of three asymmetric centers, Epitalon exists in the form of eight stereoisomers. While the naturally occurring tetrapeptide consists solely of L-amino acids, a good research direction would be to evaluate the pharmacological properties of the other seven stereoisomers in a comparative study.

## 6. Conclusions

In June 2025, it will have been 25 years since the synthesis of Epitalon was patented. During this quarter-century, multiple in vitro, in vivo, and in silico studies, as well as clinical trials on this tetrapeptide, have been conducted. However, despite the fact that many experiments have shown statistically significant geroprotective and neuroendocrine effects of Epitalon, resulting from its antioxidant, neuroprotective, and antimutagenic effects, its mechanism of action remains unclear. While it has been proven that Epitalon exhibits inter alia direct effects on melatonin synthesis, affects the mRNA levels of interleukin-2, modulates the mitogenic activity of murine thymocytes, and increases the activity of multiple enzymes such as AChE, BuChE, and telomerase, it is currently not known if these are the only routes of action of this compound. In addition, despite the relatively large number of studies on the biological and pharmacodynamic properties of Epitalon, the number of physico-chemical and structural works on this peptide are very limited. Since the presence of this compound has been recently confirmed in pineal gland polypeptide complex solution, this additionally justifies the necessity of further studies on this unusual tetrapeptide.

## Figures and Tables

**Table 1:** Cell lines and corresponding experiments performed with them mentioning the influence of Epitalon.

Cell Type/Line	Origin	Parameter Investigated	Reference
Isolated primary retinal cells	Rat	Proliferation and growth dynamics	[17]
Isolated pigmented epithelial cells			
Isolated hepatocytes	Rat	Protein synthesis	[15]
Isolated pinealocytes	Rat	Transcription factor and enzyme level	[22]
		Expression of CD5, CD20, CD4, and CD8	[24]
Perifused pineal gland	Rat	Melatonin release	[23]
Isolated primary fibroblast	Rat	Expression of Ki-67, CD98hc, caspase-3, and MMP-9	[53]
Organotypic cultures of isolated skin explants	Rat	Ratio of the total explant area	[55]
Brain cortex tissue	Rat	Proliferation	[14]
Subcortical (pineal) tissue			
Liver tissue			
Thymus tissue			
Bone marrow tissue	Mouse	SPC-F and CD4+ cells population	[44]
Splenocytes		IL-2 mRNA expression	[26]
		Contribution of CD8+ cells	[44]
Isolated peritoneal macrophages	Mouse	Lymphocyte-activating characteristics of macrophages	[47]
HeLa	Human	Telomerase subunit expression and telomere elongation	[33]
		Membrane penetration	[41]
		DNA binding	[41]
Fetal lung fibroblasts 602/17	Human	Telomerase subunit expression and telomere elongation	[33]
		Division limit	[34]
Thymocytes	Mouse	Proliferation	[28,29,31]
		Neutral sphingomyelinase activity	[31]
		Thymic serum factor presence in supernatant fraction	[44]
		Involvement in sphingomyelin pathway	[28]
	Human	Argyrophyllic proteins expression	[38]
		Aging, L7A protein expression	[39]
Pinealocytes	Human	Aging, L7A protein expression	[39]
Thymic epithelial cells	Human	Argyrophyllic proteins expression	[38]
Isolated lymphocytes	Human	Effects on chromatin	[35,36,37]
		Telomere length and mitotic index	[25]
NB7 neuroblastoma	Human	NEP and IDE expression	[27]
Fission cavity	Frog ^1^	Differentiation	[32]
SH-SY5Y neuroblastoma	Human	AChE and BuChE activity	[45]
		Amyloid precursor protein secretion level	[45]
THP-1 monocytic leukemia	Human	Inflammatory pathways	[46]
Fibroblasts derived into neurons	Human	DNA damage prevention, mitochondrial and lysosomal activity, and morphology post-Epitalon administration	[50]
Periodontal ligament stem cells	Human	MTT, GAP43, and Nestin protein expression	[16]
		p16 and p21 expression	[52]
MII oocytes	Mice	ROS level, morphology, mitochondrial membrane potential, mtDNA copy number, apoptosis, and DNA damage	[51]
Gingival mesenchymal stem cells	Human	GAP43, Nestin, β-tubulin III, and Doublecortin mRNA expression	[40]
		p16 and p21 expression	[52]
Retina	Chicken	Pax6, Vsx1, Brn3, Prox 1, and TTR expression	[21]
Isolated skin fibroblasts	Human	SOD-1, NQO1, and catalase expression	[54]
Cumulus cells	Bovine	Telomerase activity, mitochondrial health, and mRNA expression	[56]
Oocytes		Telomerase activity, TERT protein localization, and mitochondrial health	
Post-thawed embryos		Development, re-expansion, implantation potential, mitochondrial health, and trophectoderm integrity	

^1^ Xenopus laevis.

**Table 2:** Mice strains and corresponding experiments performed with them mentioning the influence of Epitalon.

Strain	Route of Administration	Dose	Period of Administration	Parameter Investigated	Reference
SAMP-1	Subcutaneous injection	1 µg/mouse, 5 times a week	10 months	Incidence of chromosome aberrations	[62]
SAMR-1	Subcutaneous injection	1 µg/mouse, 5 times a week	10 months	Incidence of chromosome aberrations	[62]
SHR	Subcutaneous injection	1 µg/mouse, 5 times a week	10 months	Incidence of chromosome aberrations	[62]
			9 months	Incidence of chromosome aberrations in bone marrow	[63]
			Until natural death	Body temperature, life span, food consumption, estrous function, and weight	[63]
CBA	Subcutaneous injection	0.1 µg/mouse, 5 times a week	Until natural death	Body weight and food consumption dynamics, physical activity, age-related estrus functionality, body temperature, muscular strength and physical fatigability, longevity, and carcinogenesis	[65]
			1 day	Free radical processes	[65]
		1 µg/mouse, everyday	5 days	Gene expression in heart	[74]
		0.1 µg/mouse, everyday	5 days	Gene expression in brain	[75]
			5 days	Antioxidant properties, superoxide dismutase activity	[11]
HER-2/neu	Subcutaneous injection	1 µg/mouse, 5 times a week	Until natural death	Longevity of mice and incidence and count of new breast tumors	[66,68,72]
				Tumor parameters, HER-2/neu mRNA expression	[67,68]
				Β-actin expression in mammary tumors	[67]
				Stressor influence	[72]
		1 µg/mouse, 5 consecutive days every month		Life span, stressor influence, longevity of mice, incidence, and count of new breast tumors	[72]
C3H/He	Subcutaneous injection	0.1 µg/mouse, 5 times a week	6.5 months	Incidence, localization, and type of tumors	[71]
No data/no strain	Subcutaneous injection	5 µg/kg	Single dose	Anomalies in sperm heads, presence of micronuclei in erythrocytes of peripheral blood, and hair color dependence	[73]

**Table 3:** Rat strains and corresponding experiments performed with them mentioning the influence of Epitalon.

Strain	Route of Administration	Dose	Period of Administration	Parameter Investigated	Reference
Wistar	Subcutaneous injection	0.5 µg/rat	30 days and 42 days	Three gastric endocrine occurrence after pinealectomy	[78]
			10 days	Functional spleen morphology of post pinealectomy rats	[10]
		5 µg/rat		Pineal gland morphology after γ-irritation	[87]
	Intraperitoneal injection	0.5 µg/rat	5 days	Splenic lymphocytes apoptosis after γ-irritation	[86]
		5 µg/kg		Post γ-irritation morphology, proliferative activity, and immunohistochemical investigation of spleen, thymus, and duodenum	[79]
		2.5 µg/kg		Lipid peroxidation in brain and serum	[1]
		4 µg/kg	4 days	Noradrenaline and dopamine in proestrus regulation	[99]
	Intramuscular injection	2 µg/rat	Single dose	Number and optical density of IL-2-positive cells in hypothalamic structures	[97]
	Intranasal	2 µg/rat			
		0.5 µg/rat	Every 12 h (2 days)	C-Fos protein content in pineal gland	[89]
		20 ng/rat	Single dose	Cortical neuron activity	[100]
				C-Fos and IL-2 content in hypothalamic structures	[90]
	Orally	100 mg/rat	14 days	Body weight and activities of membrane-bound digestive enzymes	[92]
		100 µg/rat	1 month	Body weight and activities of subepithelial membrane-bound digestive enzymes	[93]
	Microinjection into the intercellular medium of neocortex	0.5 µL of 10^−11^ M	Single dose	Cortical neuron activity	[100]
	No data	2 µg/kg	4 days	Influence on premature aging of reproductive functions	[98]
LIO	Subcutaneous injection	0.1 µg/rat; 5 times a week	Until natural death	Influence on tumor occurrence and lifespan in different light exposure schemes	[84,85]
		1 µg/rat (0.1 mL)	5 days a week, until natural death	Age-related changes in the estrous cycle in different lighting conditions	[98]
			5 days a week, 6 months	Colon cancer treatment and apoptosis index	[81]
				Chemically induced colon carcinogenesis incidence	[80]
Campbell	Parabulbar injection (both eyes)	1 µg/rat (0.2 mL)	72 days (starting from birth)	Retina morphology and electroretinogram measurements	[18]
		0.2 µg/rat (0.2 mL)			[20]
	Intraperitoneal injection	(1, 3) 1 µg/rat (2) 0.5 µg/rat	(1) 3 weeks before mating and during pregnancy; (2) days 5–35 of life; and (3) day 35–81 of life	Retina morphology and electroretinogram measurements	[19]
Sprauge Dawley	Intramuscular injection	10 µg/kg	Single dose	IL-2 mRNA gene expression in hypothalamus	[96]
	Intranasal	1.5 or 10 µL of 10 ng/µl			
No data/no strain/albino	Orally	100 µg/rat	1 month	Body weight, length of small intestine, and passive and active glucose and glycine transport	[13]
	Intraperitoneal injection	7 µg/rat	10 days	Morphology and functionality of kidneys	
	Subcutaneous injection	0.1 µg/rat; 5 times a week	2, 8, or 14 months	Pepsin activity and total proteolytic activity	[82]
	Intramuscular injection	7 μg/kg	7 days	Kidney enzyme activity	[102]
	Intraperitoneal injection	7 μg/kg	7 days	Kidney functionality after cis-platin-induced kidney failure	[103]

**Table 4:** Epitalon docked to amino acid transporter protein ICM-Score obtained by molecular modeling.

Receptor Protein	PDB ID	ICM-Score
LAT1-4F2hc	6IRT	−32.93
LAT2-4F2hc	7CMH	−23.62
Apo HsPepT1 (PEPT1)	7PN1	−18.00

## Data Availability

Not applicable.
